# Comparative Evaluation of Light-Trap Catches, Electric Motor Mosquito Catches and Human Biting Catches of *Anopheles* in the Three Gorges Reservoir

**DOI:** 10.1371/journal.pone.0028988

**Published:** 2012-01-03

**Authors:** Wang Duo-quan, Tang Lin-hua, Gu Zhen-cheng, Zheng Xiang, Yang Man-ni, Jiang Wei-kang

**Affiliations:** National Institute of Parasitic Disease, Chinese Center for Disease Control and Prevention, WHO Collaborating Center for Malaria, Schistosomiasis and Filariasia, Shanghai, China; Weill Cornell Medical College, United States of America

## Abstract

The mosquito sampling efficiency of light-trap catches and electric motor mosquito catches were compared with that of human biting catches in the Three Gorges Reservoir. There was consistency in the sampling efficiency between light-trap catches and human biting catches for *Anopheles sinensis* (r = 0.82, P<0.01) and light-trap catches were 1.52 (1.35–1.71) times that of human biting catches regardless of mosquito density (r = 0.33, P>0.01), while the correlation between electric motor mosquito catches and human biting catches was found to be not statistically significant (r = 0.43, P>0.01) and its sampling efficiency was below that of human biting catches. It is concluded that light-traps can be used as an alternative to human biting catches of *Anopheles sinensis* in the study area and is a promising tool for sampling malaria vector populations.

## Introduction

The Three Gorges Reservoir is located at North latitude 29°16′∼31°50′, East longitude 106°20′∼110°30′ [1]. The area had a history of falciparum malaria and vivax malaria epidemic, while the transmission vectors were only *Anopheles sinensis* with the density peaking from June to September in recent years [2]. The collection of malaria mosquitoes landing on human ‘baits’ is considered the most direct and reliable method for determining human-biting activity since female mosquitoes are collected as they attempt to feed on human collectors [3]. However, the human biting catches method is labour intensive and unreliable because of variation [4]–[5] in host attractiveness of collectors. For the estimation of malaria transmission intensity, it is an important prerequisite that the sampling methods used are calibrated against the human biting catches [6]. This is because human biting catches translates directly into human biting rates, which serves as an essential parameter in the estimation of both entomologic inoculation rate and vectorial capacity [7].

Many sampling methods [8] have been evaluated as an alternative to human biting catches with varying degrees of success. The evidence [9] that light-traps can provide an estimate of human-biting activity was validated by comparison with human biting catches conducted concurrently. The work of Lines [10] suggesting that the number of *Anopheles* caught by light-traps in East Africa is proportional to that by human bait, represented an important advance. The study [11] in Lawanda village of western Kenya also indicated that despite the clear difference in the number of mosquitoes caught by each method, both the Mbita trap and light trap catches were directly proportional to human biting catches regardless of mosquito density. Above all, a series of studies [12]–[14] demonstrated that light-traps may be as a sensitive alternative to estimate human-biting activity of *Anophelines*.

On the contrary, Mbogo [15] claimed that in Kilifi, Kenya, this proportionality was not observed, and the CDC light-trap [16] hung close to a human sleeping under a bed net with an incandescent bulb, was not considered a reliable means for estimating malaria vector outdoor biting densities.

However, no studies about the comparative field evaluation of light-trap catches, electric motor mosquito catches and human biting catches had been carried out in China until now. Therefore, we report the results of a parallel series of conventional human biting catches and light-trap catches or electric motor mosquito catches, and our objective was to determine whether the light-trap catches or electric motor mosquito catches may be used in place of human biting catches to monitor the human-biting rate.

## Methods

### Study area

According to some socioeconomic factors and environmental features (e.g. pesticides use, local sleeping outdoors and mosquito net use, paddy field, riparian zones) relating to the malaria vectors distribution, four villages (Fuling, Wenzhou, Kaixian and Fengjie) were selected from different sections in the Three Gorges Reservoir region . With informed consent and active cooperation of the villagers, this study was undertaken from 2008 to 2009 in the selected villages (population 500–1000 each).

House design usually consisted of either a one- or two-room mud-daubed construction with a low, thatched roof. The eaves of most houses were open, which facilitated mosquito ingress and egress. The average family size was about five people per house, together with their chickens, often a dog, but few other livestock. Cooking occurred typically inside the homes or under the eaves of a porch. The detailed description of the study area and the maps showing the location of the selected villages are provided elsewhere [17]. Depending on geographic size, each selected village was divided into four sectors and four houses were selected randomly upon receiving consent from the household heads from every sector in the selected villages when the survey was carried out.

### Sampling method

Light-trap catches and electric motor mosquito catches were performed with the human biting catches for two consecutive nights biweekly between June and September from 2008 to 2009 in each selected village. The Light-trap catches and electric motor mosquito catches were conducted in the same houses as the human biting catches from 18:00–06:00, but were made on the night either immediately before or after the human biting catches. Four light–traps (LTS-M02, Voltage: 220V/50HZ, Motors Input: 12w, Air flow: 1.4 m/s; designated by China CDC as the tool for national mosquito surveillance) were operated outside of every chosen houses in each selected village. Each light-trap was hung about 1.5–2.0 m above the ground. Each householder participated in the study and was instructed in the proper operation of the light-trap: to turn the trap on at sunset, to close the neck of the trap collection bag at sunrise, to prevent the mosquitoes from escaping, and to turn off the motor. The traps were collected in the morning by project staff, inquiries were made as to whether the trap functioned properly all night and proper light-trap operation during the night was ensured by periodic inspection. Meanwhile, mosquitoes resting indoors of the chosen houses were collected by electric motor mosquito catches (CN85202146) by two persons at the same time as the light-trap catches. Human biting catches were carried outdoors of two chosen houses, the catches rotated through the four sections in each selected villages on different nights, and thus sampling was repeated twice in each village per month. According to WHO recommendations [18], human biting catches were made by two adult volunteers from the local population working beside the bednet with one sleeping person. Mosquitoes coming to bite the collectors or sleeping person were detected using a flashlight, collected with glass tubes (CDC backpack aspirator: John W. Hock Co., Florida, USA) and placed in the screened pint-sized containers. Collections were conducted for 30 min each hour from 18:00 pm to 06:00 am overnight. Collectors worked in pairs for 6-h shifts. One pair began at 1800 h and the other at midnight. Mosquitoes were taken to the laboratory and killed by suffocation with chloroform vapor. They were counted and identified morphologically using taxonomic keys [19].

### Ethical considerations

We have obtained ethics approval from National Institute of Parasitic Disease, Chinese Center for Disease Control and Prevention (Who Collaborating Center for Malaria, Schistosomiasis and Filariasis) ethical committee and written informed consent was obtained from all the participants. No specific permissions were required for these activities, the location is not privately-owned and the field studies did not involve endangered or protected species.

### Statistical methods

Exploratory analysis indicated that the data were not normally distributed and lacked homoscedasticity. To maintain the assumptions for analysis, the average numbers in each catch (x) were transformed to y = log(x+1) to normalize prior to statistical analysis. Data for the mosquitoes caught by the indoor collections of the electric motor mosquito catches and outdoor light-traps were analyzed to estimate the electric motor mosquito catches or light-traps as compared to human biting catches following similar procedures as in Lines [8]. The aims were: to establish whether the two sampling methods were correlated by calculating the Pearson correlation coefficients for the relationship among log(x+1) transformed catches of different methods; to compare the efficiency of different methods in estimating mosquitoes abundance by utilizing graphical and parametric methods of Altman and Bland [20]; and to test for the differences of sampling efficiency between months, villages by the statistical comparison (ANOVA) of the corresponding methods.

## Results

Overall, our study was carried out in the Three Gorges Reservoir in four representative villages for 256 nights distributed over sixteen months, with a final comparison between 256 nights for light-trap catches or 512 men-nights for electric motor mosquito catches and 512 men-nights for human biting collections. The number of mosquitoes collected by the light-trap catches, electric motor mosquito catches and the human biting catches method were 6610, 680, and 1640 *Anopheles sinensis* respectively. The parameter indicate that the light-trap catches, electric motor mosquito catches caught about 403% and 41% of the number of *Anopheles sinensis* caught in the human biting catches.

**Figure 1:**
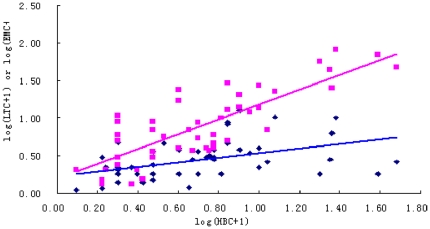
Scatter distribution for the relationship between light-trap catches (LTC) (red color) or the electric motor mosquito catches (EMC) (blue color) and the human biting catches (HBC) of *Anopheles sinensis* (Logarithmic sclaes).

Further, scatter distribution for the relationship between light-trap catches or the electric motor mosquito catches and the human biting catches (Fig. 1) indicated that there was consistency in the sampling efficiency between the light-trap catches and the human biting catches for *Anopheles sinensis* (r = 0.82, P<0.01), while the correlation coefficient between the electric motor mosquito catches and the human biting catches was found to be not statically significant (r = 0.43, P>0.01).

One might wish to go further, and predict what the human biting catches would have been on the night of a given light-trap catches. Altman & Bland [20] have pointed out that making such predictions from a linear regression may be misleading. Instead, the ratios between two types of catches against the geometric mean of the two catches were used as a measure of their relative sampling efficiency.

**Figure 2:**
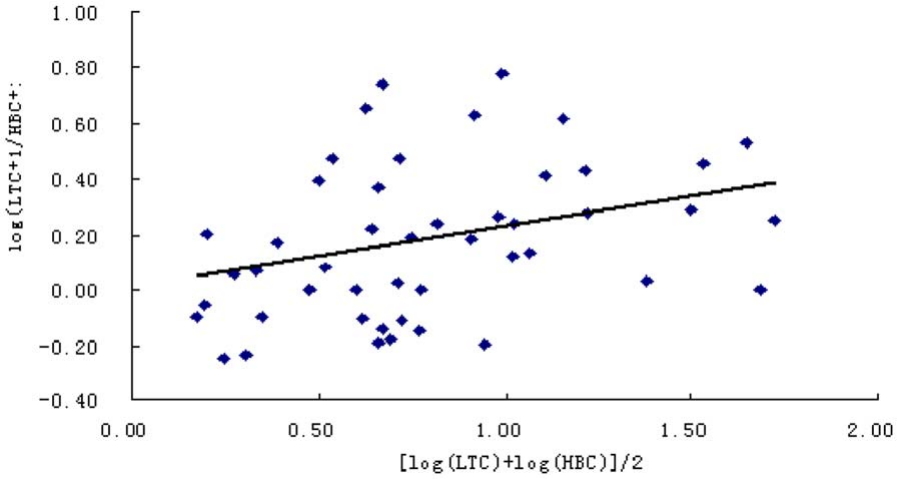
The relationship between light-trap catches (LTC) and the human biting catches (HBC).

There was no significant tendency for the ratio of light-trap catches to human biting catches to increase with increasing mosquito abundance (r = 0.33, P>0.01), Fig. 2 also shows that the vertical scatter of the observations (i.e., the variance of the log-ratios) shows little or no relationship to mosquito density which means that the variability of the ratio between the catches varied independently of changes in mosquito density. The mean log ratio was 0.1816 (s.e. 0.026). Taking the antilog gives the geometric mean ratio, 1.52 (95% confidence interval 1.35 to 1.71). This means that on average, the catches from the light-traps was 1.52 times that from the human biting catches, and 95% of light-trap catches are expected to lie from 0.35-fold to 0.71-fold greater than the catches with two human collectors outdoors.

Analysis of variance was used to test whether the relative sampling efficiency of the two types of catches varied according to months, or to the villages where the catches were made. No significant biases were found among the months, while significant difference existed in the villages (table 1).

**Table 1:** Analysis of variance on the log-transformed ratios between the light-trap catches (LTC) and the human biting collections (HBC), calculated as log [(LTC + 1)/(HBC+ 1)].

	d.f	Sum of Squares	Mean Square	F	P
Months	3	0.32	0.11	3.71	P>0.01
Villages	3	1.78	0.59	20.91	P<.001

**Figure 3:**
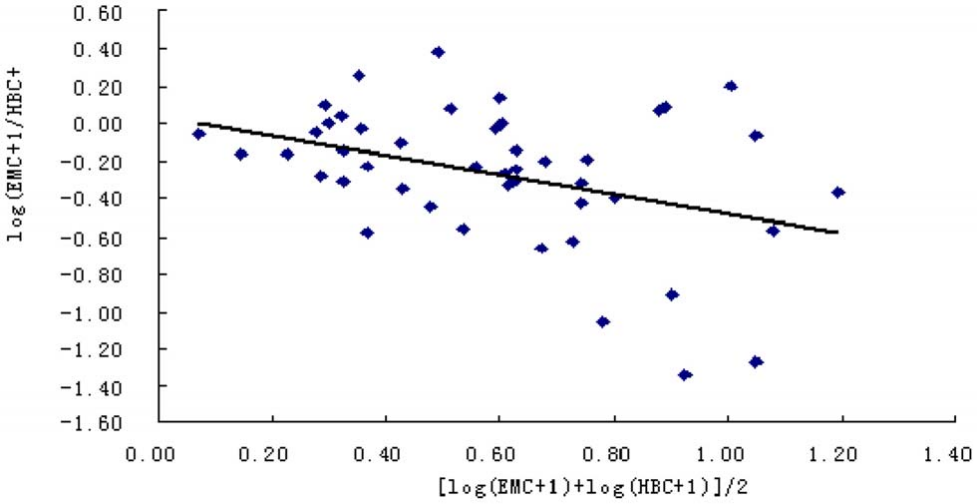
The relationship between the electric motor mosquito catches (EMC) and the human biting catches (HBC).

On the contrary, no significant tendency for the ratio of EMC/HBC to increase with increasing mosquito abundance (r = −0.40, P>0.01), while the Pearson correlation coefficient was negative (Fig. 3) which means the sampling efficiency of EMC was below that of HBC at high density.

## Discussion

Both the electric motor mosquito catches and the light-trap catches were intended to provide estimates of the density of human-biting mosquitoes in the trial villages. This study demonstrated that light-trap catches can be used as an alternative to human biting catches of *Anopheles sinensis* in the study area. The evidence that light-trap catches can provide an estimate of human-biting activity was validated by comparisons with human biting catches. We found that light-trap caught 1.52 times the number of *Anopheles sinensis* as captured by human biting catches which indicate that light-traps may be a sensitive means to estimate human-biting activity of *Anopheles sinensis* in this area. Further, the relationship was not affected significantly by changes in the mosquito density, date of sampling the mosquitoes in the study area. Though some studies [21]–[24] showed that light traps catched fewer *Anopheles sinensis* than human bait, our findings was in agreement with some studies [10], [25]–[26] that they catched more mosquitoes than human biting catches. This difference of sampling efficiency may be related to some differing epidemiologic settings, such as the differences in sleeping arrangements, availability of alternative hosts, temperatures, humidity, and wind speed. There is, therefore, a need to standardize the operational conditions and sampling procedures used if valid comparisons between various studies are to be made.

However, the correlation between the electric motor mosquito catches and the human biting catches was not statistically significant, though no density-dependent sampling efficiency was noted for the comparison, their correlation coefficient indicated that the electric motor mosquito catches was less efficient than the human biting catches for sampling *Anopheles sinensis* at high density in the study area.

Several factors might explain this observation. First, the engorged mosquitoes may escape before being trapped indoors. Second, the malaria vectors in this area are dominated by Anopheles sinensis , a mosquito species that is usually largely exophilic but zoophagic. Third, a questionnaire administered to the study villages indicated that more than one pesticide was applied indoors in every family on hot days. In addition, some residents like to sleep outdoors without protections.

This is the first study for evaluating the performance of the light trap catches relative to the human biting catches for *Anopheles sinensis* in China, additional field trials are needed to determine whether the relationship between light-trap catches and human biting catches are influenced by differences in host preference, feeding behavior among different epidemiologic settings so that its flexibility and consistency can be fully explored prior to wider application. However, the human biting catches should be maintained as the standard reference method, it is important to perform limited number of human biting catches to re-check whether the light-traps can be relied upon to provide an unbiased measure of density at each new location. There is therefore a need to standardize the use of this method to enable valid comparisons of results from the various studies in different epidemiologic settings.

Nevertheless, at this stage it can be argued that the light trap catches is a promising tool for sampling malaria vector populations and may be very useful for enabling community members in collecting large numbers of samples that are representative of the overall vector population at a less cost.
